# Rapid, automated imaging of mouse articular cartilage by microCT for early detection of osteoarthritis and finite element modelling of joint mechanics

**DOI:** 10.1016/j.joca.2014.07.014

**Published:** 2014-10

**Authors:** P. Das Neves Borges, A.E. Forte, T.L. Vincent, D. Dini, M. Marenzana

**Affiliations:** † Department of Bioengineering, Imperial College London, London SW7 2AZ, UK; ‡ Department of Mechanical Engineering, Imperial College London, London SW7 2AZ, UK; § Kennedy Institute of Rheumatology, University of Oxford, Oxford OX3 7HE, UK

**Keywords:** Mouse articular cartilage, Destabilisation of medial meniscus model, Micro computed tomography, High-throughput automated image analysis, 3-dimensional quantitative imaging

## Abstract

**Objective:**

Mouse articular cartilage (AC) is mostly assessed by histopathology and its mechanics is poorly characterised. In this study: (1) we developed non-destructive imaging for quantitative assessment of AC morphology and (2) evaluated the mechanical implications of AC structural changes.

**Methods:**

Knee joints obtained from naïve mice and from mice with osteoarthritis (OA) induced by destabilization of medial meniscus (DMM) for 4 and 12 weeks, were imaged by phosphotungstic acid (PTA) contrast enhanced micro-computed tomography (PTA-CT) and scored by conventional histopathology. Our software (Matlab) automatically segmented tibial AC, drew two regions centred on each tibial condyle and evaluated the volumes included. A finite element (FE) model of the whole mouse joint was implemented to evaluate AC mechanics.

**Results:**

Our method achieved rapid, automated analysis of mouse AC (structural parameters in <10 h from knee dissection) and was able to localise AC loss in the central region of the medial tibial condyle. AC thickness decreased by 15% at 4 weeks and 25% at 12 weeks post DMM surgery, whereas histopathology scores were significantly increased only at 12 weeks. FE simulations estimated that AC thinning at early-stages in the DMM model (4 weeks) increases contact pressures (+39%) and Tresca stresses (+43%) in AC.

**Conclusion:**

PTA-CT imaging is a fast and simple method to assess OA in murine models. Once applied more extensively to confirm its robustness, our approach will be useful for rapidly phenotyping genetically modified mice used for OA research and to improve the current understanding of mouse cartilage mechanics.

## Introduction

Cartilage pathogenesis in osteoarthritis (OA) has been shown to be driven by common molecular players shared by human and mouse and, thanks to their amenability to genetic studies, murine models of OA are becoming essential tools for drug discovery^1^. Mouse models of OA include spontaneously occurring OA, as in the Str/ort strain^2^, and surgically-, chemically- or overloading- induced OA^3^. The main limitation of mouse models is that, due to the limited size of their joints, it is not possible to use established non-invasive imaging technologies (e.g., quantitative computed tomography, QCT, and magnetic resonance imaging, MRI) used in humans to diagnose OA^4^, ^5^, ^6^. Currently, most research labs use conventional histopathology scoring to assess the articular cartilage (AC) in the mouse knee. Besides limiting the translational potential, this makes the murine models extremely time consuming, low throughput and costly^3^.

Efforts by different labs to develop non-destructive (*ex vivo*) imaging methods to assess mouse AC more efficiently have multiplied in the past 5 years^7^, ^8^, ^9^, ^10^. The benefits of these efforts include 3D-dimensional (3D) visualisation of the mouse AC surface and computation its volume, which have allowed localising OA lesions and apply quantitative approaches to the determination of OA severity.

Micro-computed tomography (microCT) is a high-resolution, non-destructive, 3D imaging technology which revolutionised bone research in small rodents^11^ and was recently employed to quantify changes in subchondral bone (SCB) in rodent models of OA^12^. Using radiopaque staining agents, microCT can be used also to visualise soft tissue with high accuracy^13^, ^14^, ^15^. Ionic partition contrast agents (e.g., ioxaglate) have been used to visualise AC and estimate sulphated glycosaminoglycans (sGAGs), content in small rodent models of arthritis^8^, ^16^, ^17^, ^18^. Whilst providing useful compositional information, with the potential to be used diagnostically in the clinic^19^, ^20^, these methods are affected by a variable cartilage contrast (depending on composition), which limits their applicability to determine AC structure. Alternatively, mouse AC and SCB were successfully visualised in 3D at multiple scales, using special fixation/staining protocols combined with new generation, phase-contrast X-ray microCT^9^. New lab-based non-commercial systems exploiting phase-contrast X-ray microCT have opened the possibility to image mouse cartilage without the need of contrast agents^21^ but will require further development. However, in all the above-cited imaging methods, time consuming manual contouring had to be employed for the assessment of mouse AC in microCT datasets. This greatly limits the throughput of the analysis and is affected by the imprecision naturally associated with manual operations and by subjective interpretation of the anatomy.

Originally used as mordant for histological staining^22^ and subsequently as heavy metal tissue stain for electron microscopy^23^, phosphotungstic acid (PTA) has been lately shown to be an excellent contrast agent to image soft tissue by microCT^14^, ^15^. PTA reveals preferentially collagenous structures^24^, although the binding mechanism is not fully understood^25^, ^26^.

In the current study, we tested whether PTA would provide sufficient contrast enhancement, for conventional microCT imaging, to visualise in 3D and automatically segment mouse AC.

Although altered mechanics is a recognised factor in OA pathogenesis^27^, the mechanical characterisation of the mouse joint has been surprisingly neglected. Due to its limited size and thickness, the mechanical response of mouse AC is extremely difficult to test empirically. Finite element (FE) models allows *in silico* evaluation of stresses and displacements at the joint interface with very high spatial resolution. The relationship between the local mechanical response of AC and the progressive structural damage induced by OA pathology can therefore be explored. However, the construction of realistic (FE) models requires accurate 3D morphology of the mouse AC, which is currently not available. Here, we demonstrate that automatically segmented datasets of the mouse knee, including cartilage and its mineralised support, can be used to rapidly build FE models, thereby achieving high-throughput biomechanical characterisation of mouse AC.

## Method

### Animals and destabilisation of medial meniscus surgery

The work was conducted in the UK according to the Animals Scientific Procedures Act (1986) and was subject to both local ethical review and UK Home Office regulations. Male C57BL/6 mice (10 week old) were purchased from Charles River (UK), housed in cages on a 12-h light/dark cycle and allowed food and water ad libitum. Thirteen mice underwent surgical destabilization of medial meniscus (DMM) on the right knee, while the left knee was used as contralateral control (CTRL)^28^. Mice were euthanized 4-weeks (*n* = 9 per group) and 12-weeks (*n* = 4 per group) post-surgery. A group of naïve mice (*n* = 9) was used to assess the dynamic uptake of PTA in AC (*n* = 3) and AC structure before DMM surgery (baseline, *n* = 6). Additional seven naïve mice were used for testing the sensitivity to artificial lesions on AC (see details below). All naïve mice were euthanized at 10 weeks of age. Hind limbs were dissected and fixed for 24 h in 10% buffered formalin and subsequently stored in 70% ethanol.

### PTA contrast enhanced microCT (PTA-CT) imaging

Before imaging, fixed knee joints were further dissected under a dissection microscope to disarticulate them, carefully remove the menisci and expose the AC of the tibia. Tibiae were incubated in 1% PTA solution at room temperature for 24 h and scanned in a microCT scanner (SkyScan 1172) within the same solution. The 24 h incubation time was determined after uptake experiments from 0.5 to 72.5 h. Image acquisition (resolution 5 μm/pixel) required 35 min (parameters: 50 kV, 200 μA, 0.5 mm aluminium filter, 180° scan). Image reconstruction required 20 min (NRecon software, SkyScan). X-ray attenuation is reported in Hounsfield units (HU). After imaging, samples were washed in 70% ethanol (to remove PTA) and stored in 70% ethanol for histomorphometry.

### *Ex vivo* lesions for method validation

(1) Mechanical damage

AC was scarified by a surgical scalpel to cause artificial lesions on either medial or lateral sides (*n* = 2).

(2) Papain digestion *ex vivo*

Tibiae were incubated in papain solution (125 μg/mL papain, 0.005 M HCl, 0.1 M sodium phosphate, pH = 6.2)^29^, digested for 12 h at 60°C, washed, fixed and stored as detailed above (*n* = 2).

(3) Chondroitinase ABC (ChABC) digestion *ex vivo*

Right tibiae were immersed in 5 ml of ChABC solution prepared (0.1 U/ml of ChABC in 50 mM Tris, 60 mM NaOAc, 0.02% BSA, pH 8.0) and incubated at 37°C degrees for 8 h to induce proteoglycan depletion^17^, Left tibiae (contralateral) were kept in cold phosphate buffered saline solution (PBS). After the 8 h incubation, both digested and contralateral tibiae (*n* = 3) were fixed and stored as detailed above.

### Automated analysis of PTA-CT images

**Fig. 1:**
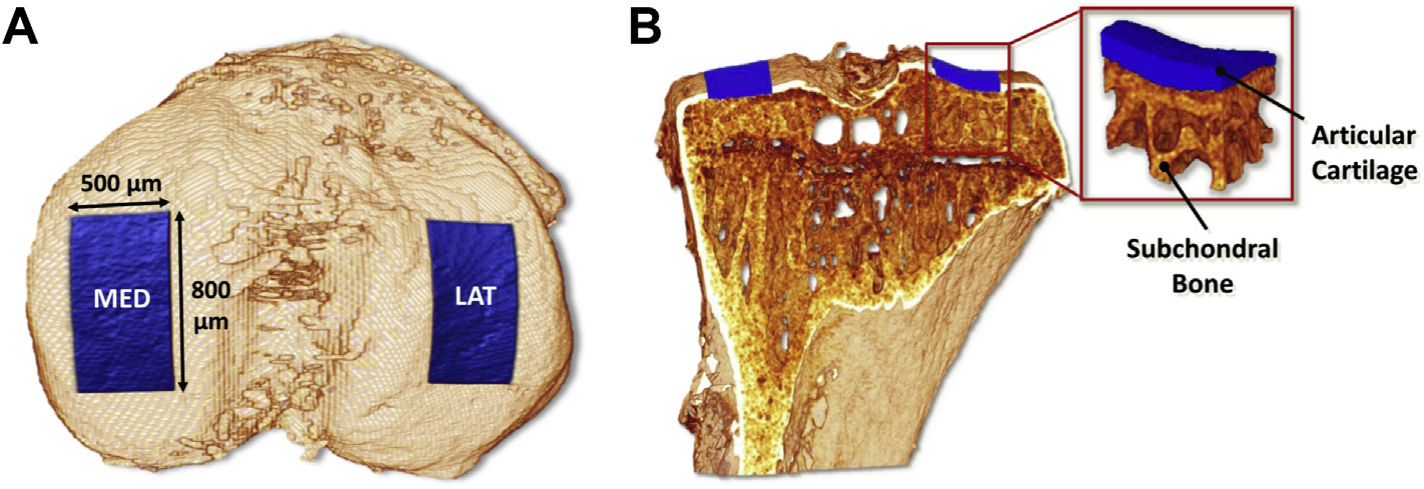
Automated mapping of ROIs generated by Matlab software on the load bearing regions on the tibial condyles. A, Top view of a 3D rendering of mouse tibia obtained by PTA-CT showing the ROIs in the medial and lateral condyles of the tibia, extending 800 μm by 500 μm. B, Coronal view of (A) and magnification of one ROI including the AC layer (coloured in blue) and extending down to the underlying subchondral mineralised plate (made of calcified cartilage and cortical bone) and subchondral trabecular bone.

Reconstructed datasets were re-sliced coronally. AC was segmented automatically using the Otsu's method which determines the optimum threshold separating two classes of grey levels (foreground and background (BKG)) as the value that minimizes intra-class variance^30^. A software utility (Matlab, MathWorks, USA) was developed to place automatically two regions of interest (ROIs), on the medial and lateral condyle of the tibia, centred on the mid coronal axis [Fig. 1(A)]. These ROIs, 500 μm wide and 800 μm long [Fig. 1(A)], were positioned using the top edge of the tibia (detected by an edge detection algorithm) and the symmetry of the condyles around the tibial central antero-posterior axis. The volume contained in the ROIs [Fig. 1(B)] was measured counting the voxels, while the average thickness of such volume was computed as the average diameter the best-fitting set of spheres (routine ‘Thickness’ in the public domain plugin for ImageJ software called BoneJ^31^). Intra-samples reproducibility was coefficient of variation (CV) = 6.4% (obtained from the same sample scanned and analysed three times) and inter-samples was CV = 8.5% (evaluated from six samples from naïve mice). Thickness maps of AC were generated by projecting onto a plane the local volumetric thickness values (Matlab).

### Undecalcified histology and histopathology grading

Undecalcified histomorphometry was used for validating the localisation of PTA staining in artificially damaged samples (*n* = 10) and in a part of the 4-week DMM group (*n* = 4). Tibiae were embedded in methyl methacrylate (MMA), using a previously described protocol^32^. Coronal sections (8 μm thick) were stained with light green/safranin-o or Von Kossa/safranin-o or toluidine blue.

Decalcified histomorphometry was used for conventional OA histopathology grading. Samples obtained from the DMM operated mice were decalcified in ethylenediaminetetraacetic acid (EDTA) and embedded in paraffin. Coronal sections (4 μm thick) were cut at regular spacing (80 μm between each level) across the joints (12 levels/joint) and stained with haematoxylin & eosin/safranin-o. Medial and lateral side were graded 0–6 by two independent assessors, using the scoring system for murine OA defined by OARSI guidelines^33^. The 2D thickness of AC in safranin-o stained sections was evaluated by manual segmentation of the AC within the ROIs described above (ImageJ).

### FE modelling of automatically segmented cartilage

PTA-CT scans of a pair of CTRL and DMM tibiae (from 4-week DMM group) and a femur and a tibia from a naïve mouse, were segmented as described above and imported in Mimics software (Materialise, Belgium). Menisci and other soft tissue elements were not included in the model. A coarser tetrahedral mesh (average element length (AEL) = 57 μm) was used for the peripheral regions away from the contact regions, while a high density tetrahedral mesh (AEL = 5.7 μm) was employed to discretise the contact areas (radius = 0.3 mm) between tibia and femur, to improve accuracy and resolution^34^ [Fig. 7(A)–(C)]. The mesh was imported in Abaqus software (Simulia, USA) to apply the required boundary conditions. The discretisation (4-node linear tetrahedron element, Abaqus C3D4) resulted in meshes of the order of one million elements for the tibiae, with over 300,000 elements assigned to the AC. A step-loading event was modelled with the joint bent at 80° flexion [Fig. 7(A)], which is the approximate flexion observed at the beginning of the gait in small rodents and when maximum contact pressure is expected in the load-bearing regions of the joint^35^. Tibia and femur were used to apply boundary conditions and for monitoring force and tied to two rigid holders placed far away from the contacting surfaces, thus not affecting AC response. These rigid holders were as we evaluated the contact pressure at the tibia–femur interface in steady-state scenario, a *static* step was set up. AC and bone were modelled as isotropic linear elastic materials (respectively, elastic modulus *E* = 6 MPa/Poisson ratio *ν* = 0.49^36^, and *E* = 18 GPa/*ν* = 0.3^37^), which is valid only in first approximation but appropriate in the context of this work, where a comparative analysis is performed based on the change in morphology rather than in the material characteristics of the tissues under consideration. The tibia holder was completely fixed while a vertical displacement was imposed to the femur holder in order to push it toward the tibia. A displacement of 0.05 mm was applied to induce loads comparable to those observed *in vivo*, while the vertical reaction force on the femoral holder was monitored. A load of 0.6 N (approximately ten times the load imposed by 25 g mouse on a single leg) was chosen to compare the three models. Each simulation required 8 h per sample.

**Fig. 2:**
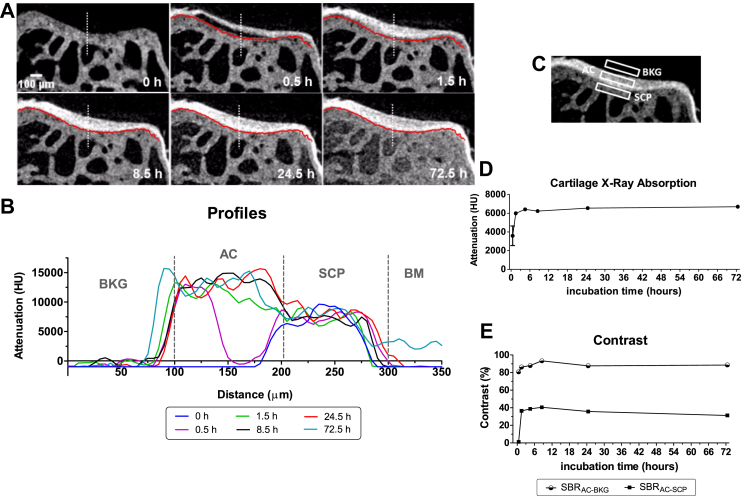
A, Representative coronal views of the lateral condyle of a mouse tibia imaged by microCT showing the uptake of PTA over time (*n* = 3). The **red line** contouring the tidemark (uncalcified–calcified cartilage boundary) was manually drawn on the first time point (at 0.5 h) and copied across on all subsequent time points to better visualise PTA diffusion beyond the tidemark. The leftmost panel shows the same view of the lateral condyle before PTA was added in the sample holder within the microCT scanner. Note that soft tissue is undetectable without contrast agent. B, Representative profiles of grey levels along the **white dotted line** shown on each coronal image in (A). Each profile represents an incubation time point (in PTA solution). Transitions between BKG and AC, AC and SCP, SCP and BM are marked on the profiles by **grey dotted lines**. C, Representative coronal view of the lateral condyle of a mouse tibia showing the three ROIs AC, BKG and SCP used to measure X-ray absorption (reported as HU levels for each ROI) and related contrasts. D, Representative time course of X-ray absorption of AC as a function of PTA incubation time (*n* = 3). E, Representative time course of SBR – a quantitative measure of the contrast achieved by an area of interest within an image – over PTA incubation time. SBR was computed for AC vs BKG (SBR_AC−BKG_) as well as for AC vs SCP (SBR_AC−SCP_). PTA uptake time course up to 72 h evaluated in one sample and up to 24 h evaluated in three samples (from naïve mice). Finally the 24 h time point uptake was evaluated in 12 samples (all the naïve samples).

**Fig. 3:**
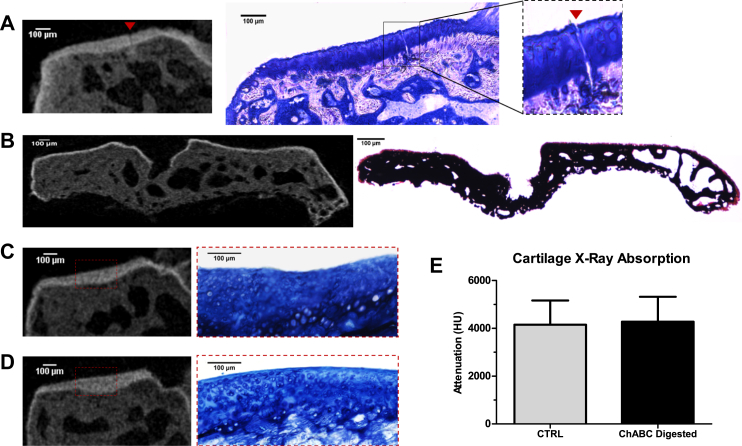
Visualisation of mechanically or chemically damaged cartilage by PTA-CT. A, Representative PTA-CT coronal view of a medial tibial condyle scarified using a surgical scalpel (*n* = 2), and correspondent histological section (undecalcified) stained with toluidine blue, showing a deep cut (**red arrow**) extending from the AC surface to the SCP. Note the diffusion of PTA below the tidemark only locally around the zone of the cut. B, Representative PTA-CT coronal view of a tibial epiphysis after 24 h digestion in papain protease (*n* = 2) and correspondent histological section (undecalcified) stained with Von Kossa/safranin-o. A thin layer of stained material above the mineralised tissue – likely to be cartilage debris of the enzymatic digestion – is visible on the surface of the SCP in both images. C–D, Representative coronal views of the medial condyles from a pair of untreated (C) or ChABC treated (D) tibiae (*n* = 3) and correspondent histological sections stained with toluidine blue. The AC of the treated samples appeared discoloured compared with untreated AC – showing the expected loss of sGAGs induced by chondroitinase digestion. However, no qualitative changes between treated vs untreated samples were observed in the PTA-CT imaging. (E) Bar graph showing the X-ray absorption of AC (expressed as mean HU level of the **red dashed ROI**) in treated and untreated groups – no statistically significant changes were found (*n* = 3).

**Fig. 4:**
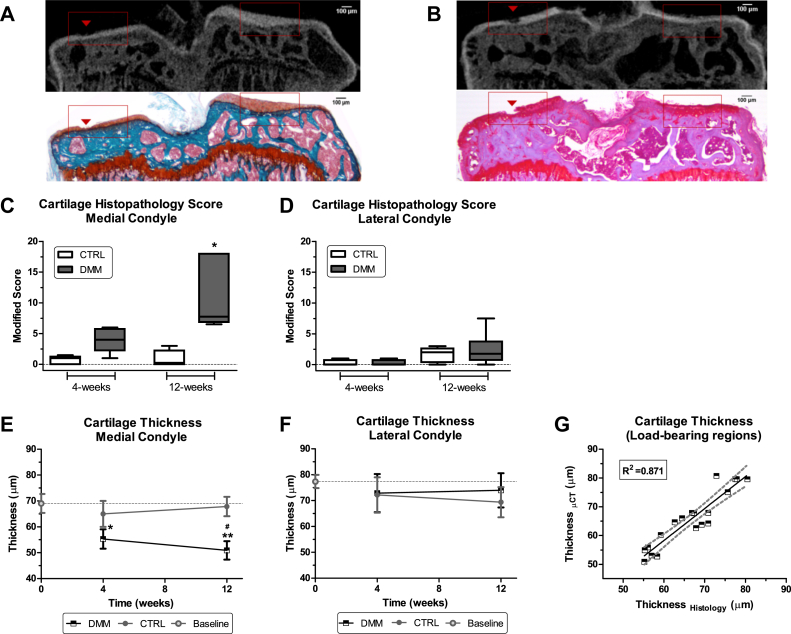
Automated assessment of AC in the DMM model and histological validation. A–B, Representative coronal views of a DMM tibia at 4 weeks (A) and 12 weeks (B) post surgery imaged by PTA-CT and correspondent histological section stained with safranin-o (contrasted with fast green in A or with haematoxylin in B). Note that a small lesions on the medial AC (**red arrow head**) are visible in the PTA-CT image and are well matched in the histological sections. C–D, Boxplot charts of the modified histopathology OARSI score for the CTRL and DMM joints at 4 and 12 weeks post DMM surgery. Data were grouped into medial and lateral. The median score of the medial side of the DMM was elevated compared with the CTRL at 4 weeks, although borderline significant (*P* = 0.06, *n* = 5, Wilcoxon signed rank test) and further increased at 12 weeks (*P* = 0.035, *n* = 4). E–F, Line charts of AC average thickness measured automatically from the volumes contained in the ROIs obtained from segmented PTA-CT scans of CTRL and DMM joints at 4 and 12 weeks post DMM surgery. Data grouped into medial and lateral. Symbols represent the means and error bars show 95% CI (*n* = 4 for experimental groups and *n* = 6 for naïve mice used as baseline). *P < 0.05 and **P < 0.01 for DMM vs CTRL by paired, two-tailed Student's *t* test; #*P* < 0.05 for 4-weeks DMM vs 12-weeks DMM by unpaired, two-tailed Student's *t* test. G, Correlation graph for AC thickness in PTA-CT and histological images (includes 4- and 12-week CTRL and DMM samples, *n* = 19). AC thickness was measured in the load-bearing regions (delimited by the **red ROIs** in A–B) from both methods. The coefficient of determination *R*^2^, obtained from the linear regression, is reported on the graph.

**Fig. 5:**
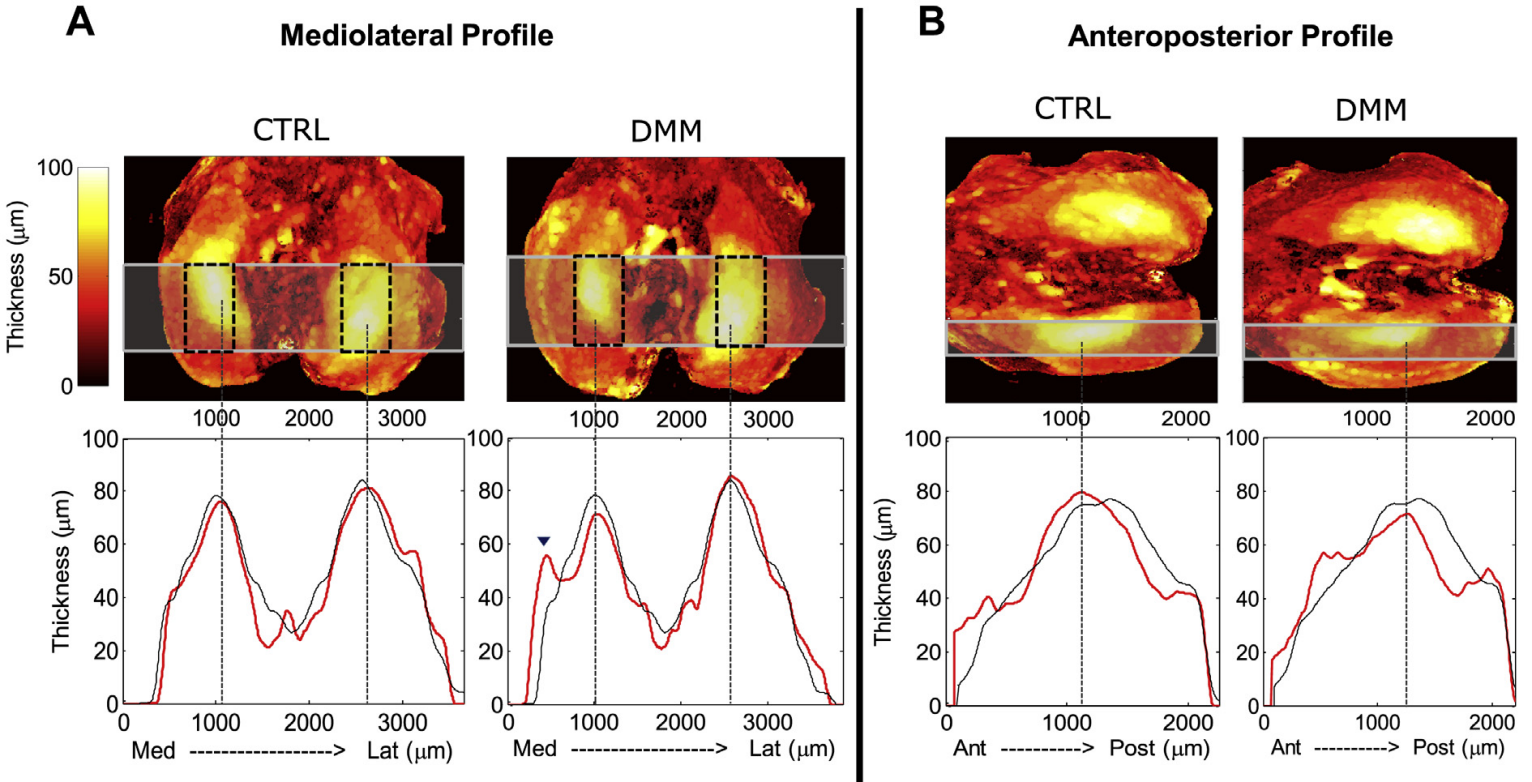
Thickness heat maps of AC obtained from PTA-CT datasets of the 4-weeks DMM group (CTRL and DMM side). A–B, Mediolateral (A) and medial anteroposterior **(**B**)** thickness maps of the AC for a representative pair of CTRL and DMM tibiae. Below each panel the **red profiles** show the changes in thickness along the horizontal bars highlighted on the maps (each profile is the average of four samples). Thickness profiles of naïve mice (*n* = 6) are displayed in black (maps not shown for naïve mice). The position of the automated ROIs on the maps is indicated by **dashed squares**. Note that the peak thickness fall in the automated ROIs and is markedly decreased 12 weeks post DMM surgery. The **arrow head** shows a second peak in AC thickness in the mediolateral DMM profile, presumably due to the newly formed cartilage covering a medial osteophyte.

**Fig. 6:**
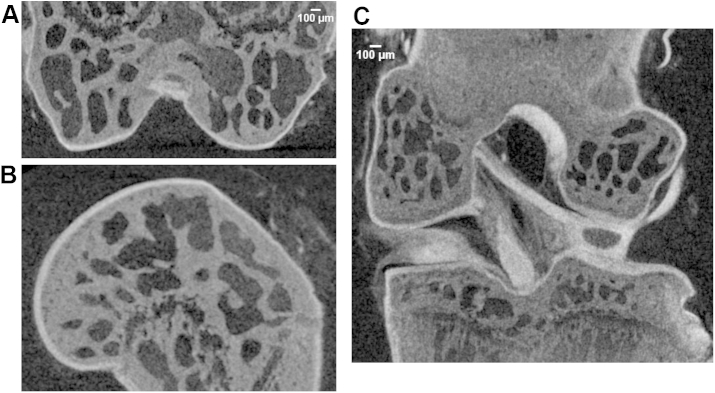
A–B, Representative PTA-CT images of a mouse distal femur displayed in coronal (A) and sagittal (B) views. C, Representative PTA-CT image showing a coronal view of a whole, intact mouse knee joint.

**Fig. 7:**
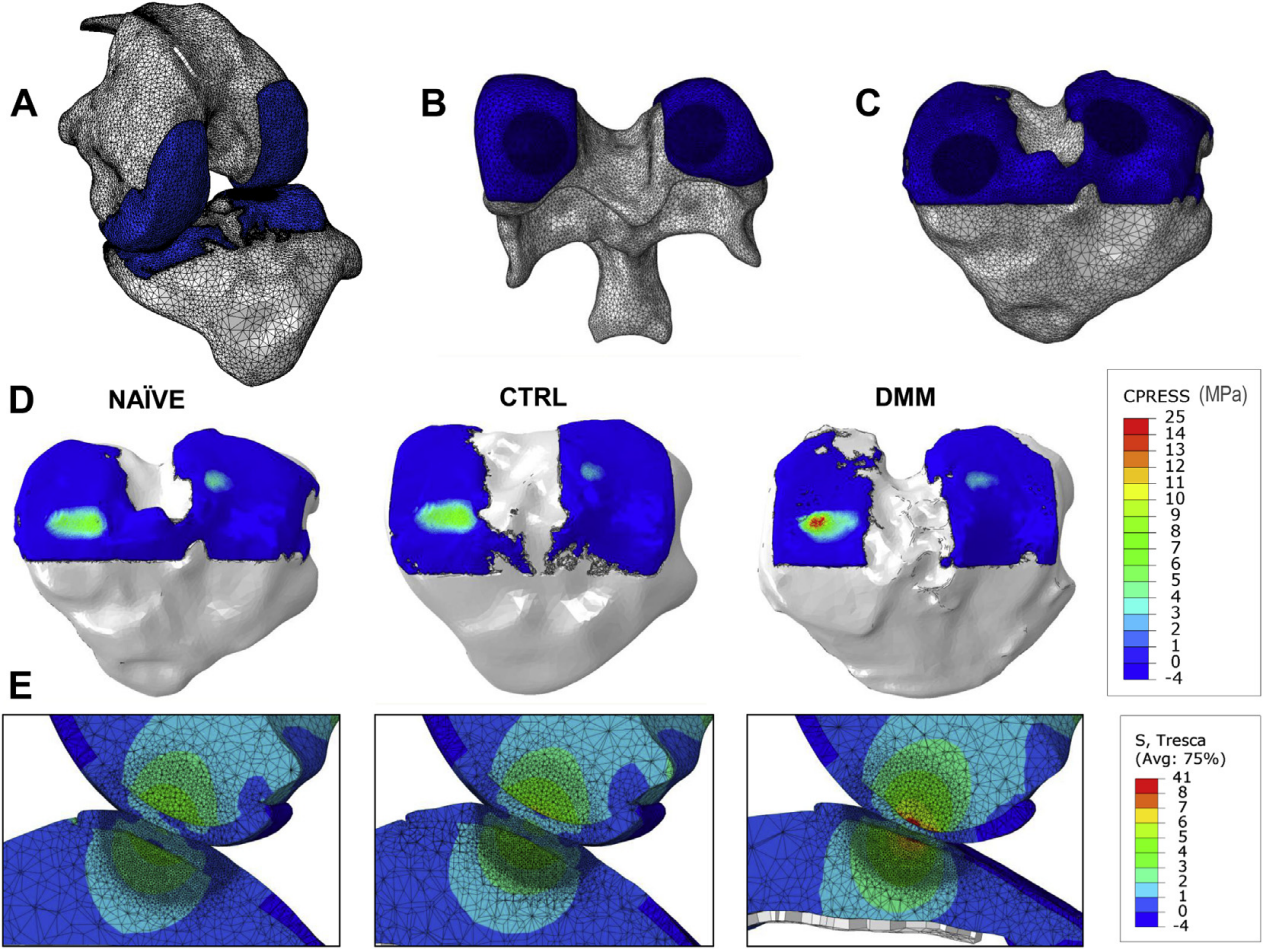
FE analysis of the mechanical stresses in AC caused by the structural changes induced by DMM surgery. A–C, model of the whole knee joint at 80° flexion (A); mesh structure and high density contact areas (darker blue) of the AC covering the (naïve) distal femur (B) and the (naïve) proximal tibia (C). D, Contact pressure maps on the surface of the AC of the three tibiae (naïve, CTRL and DMM) used in the FE simulations. E, Sagittal view of the distribution of Tresca stresses in the three modelled joints (naïve, CTRL and DMM tibiae loaded by the same naïve femur) at the end of the FE simulations.

### Statistical analysis

Data were reported as mean values ± 95% confidence intervals (CIs). For all PTA-CT data, the statistical significance of the differences between paired samples – DMM or artificially damaged vs contralaterals – were determined using a two-tail, paired Student's *t* test. Differences between the two time points were determined using a two-tail, unpaired Student's *t* test. Before applying the parametric *t* test, the near normal distribution of the data was assumed by prior knowledge and confirmed by Kolmogorov–Smirnov test which resulted non significant. Histopathology data were evaluated using paired nonparametric analysis (Wilcoxon signed-rank test). Linear regression and Pearson's correlation was computed between histomorphometry and PTA-CT parameters. Values were considered statistically different at *P* < 0.05. All statistics were computed using Prism6 software (GraphPad, USA).

## Results

### Characterisation of PTA-CT imaging

In order to measure PTA uptake into adult mouse AC, consecutive microCT scans of the same sample were acquired from time zero to 72 h. Stain-free reference images acquired prior to the uptake experiment showed no detectable cartilage layer [Fig. 2(A)]. In the presence of 1% PTA, the uptake of the stain in AC was progressive, starting from the surface at 30 min minutes (incomplete stain) and reaching the tidemark level by the 1.5 h [Fig. 2(A)]. By 1.5 h, AC appeared completely stained in the central part of the condyle but not at the margins where a less stained area could still be noted [Fig. 2(A)]. PTA uptake in AC compared with adjacent tissues were estimated by plotting the mean X-ray absorption along a line starting from the BKG space, above the AC surface, and ending in the marrow space in the SCB trabecular compartment [Fig. 2(A), (B)]. These profiles showed that AC surface was stained first and the upper edge of AC surface did not move over time. From 8 h, all profiles displayed a similar shape with a steep drop at the bottom edge of AC, i.e., at the tidemark [Fig. 2(B)]. PTA uptake dynamics was highly reproducible across the three samples tested. It is worth noting that the penetration of PTA stain through bone into the marrow was limited by the tidemark at least for up to 24 h incubation in PTA. This is supported by observing that, from 1.5 h, the X-ray absorption in the profiles fluctuated within limited bands for each distinct zone, with the exception of the bone marrow (BM) zone whose absorption increased at 72.5 h [Fig. 2(B)].

The rate of PTA uptake in AC was estimated plotting the average X-ray absorption of a ROI within AC over time [Fig. 2(C), (D)]. PTA kept accumulating at a much slower rate after 1.5 h where a plateau was reached [Fig. 2(D)].

Incubation of tibiae (not fixed) in aqueous PTA solution yielded a slower uptake and a worse contrast compared to 70% ethanol, although still sufficient for segmentation (data not shown).

The contrast of the AC layer was evaluated computing the average signal to background ratio (SBR) between a region within the AC and a region just outside AC, either in the true BKG space (SBR_AC−BKG_) or in the subchondral calcified plate (SCP) (SBR_AC−SCP_) [Fig. 2(E)]. The SBR_AC−BKG_ reached a plateau of 80–90% after the first 30 min incubation in PTA which was maintained up to 72.5 h [Fig. 2(E)]. SBR_AC−SCP_ peaked from 1.5 h incubation at ∼40% and decreased to ∼30% from 24.5 h [Fig. 2(E)]. In summary, an incubation time beyond 4.5 h and up to 72.5 h in PTA yields uniform and consistent contrast to visualise adult murine AC by microCT. We chose the 24 h incubation time point for all the following experiments mainly for historical (our initial tests) and practical reasons (experimental convenience).

### Mechanical and chemical damage of AC

A thin cut made with a surgical scalpel on the AC surface was clearly visible in PTA-CT images and was validated by toluidine blue-stained undecalcified histology [Fig. 3(A)]. Notably, an accumulation of PTA in the mineralised matrix below the cut was clearly visible. The reason for PTA leaching through the lesion is likely to be fact that the knife cut visibly broke the integrity of the tidemark which works as a barrier for PTA diffusion into the mineralized tissue. Excellent agreement between PTA-CT and histology was found for samples subjected to prolonged papain digestion. The PTA and safranin stained material (above the mineralised matrix) is likely to be a layer of debris of digested AC [Fig. 3(B)].

Incubation in chondroitinase caused GAGs loss which was revealed in the histological sections as a reduction in toluidine blue staining [Fig. 3(C), (D)] but was not detectable neither qualitatively [Fig. 3(C), (D)] nor quantitatively [Fig. 3(E)] in PTA-CT images.

### Automated assessment of OA in the DMM model

Small AC lesions at 4 and 12 weeks post DMM surgery were clearly visualised on the medial condyles of the tibiae by PTA-CT and validated by histomorphometry [Fig. 4(A), (B)]. Histopathology score increased on the medial side at 4 weeks, (*P* = 0.06) and 12 weeks (*P* = 0.035), but not on the lateral side [Fig. 4(C), (D)]. The automatically mapped ROIs included the load-bearing regions which presented the most prominent OA lesions in the DMM model [Fig. 4(A), (B)]. AC structure in 4-week and 12-week DMM groups and in naïve mice was automatically evaluated from PTA-CT scans. The average thickness of AC in DMM was increasingly reduced compared to CTRL from the 4-week to the 12-week DMM group, in the medial but not in the lateral side [Fig. 4(E), (F)]. Similar trends were found for the volume parameter and are summarised in Table I. Homologous anatomical regions (i.e., lateral or medial) did not show significant differences in CTRL compared with naïve [Fig. 4(E), (F)].

**Table I:** Relative % differences in the quantitative structural parameters of the mouse AC between paired DMM and CTRL samples in the medial and lateral condyles of the tibia at 4- and 12-week post DMM surgery. Percentage differences calculated between DMM/CTRL pairs of means; *n* = 4; *P < 0.05 for differences between 12-week medial vs 4-week medial parameters (computed by two-tails, unpaired, Student's *t* test)

PTA-CT parameters	4-weeks		12-weeks	
	Medial	Lateral	Medial	Lateral
Thickness	−14.8%	+1.2%	−24.8%*	+6.7%
Volume	−15.4%	+1.3%	−26.9%*	+11.8%

Linear regression revealed a strong correlation between 3D AC thickness from PTA-CT scans and the 2D thickness measured manually in histological sections [Fig. 4(G), Table II].

**Table II:** Correlation table. 3D thickness values measured automatically in PTA-CT scans were paired with 2D thickness values measured manually in histological sections and linear regression was computed. Data were analysed in three sub-groups including both DMM and CTRL samples: a) 4-week (*n* = 9), b) 12-week (*n* = 10) or c) all together 4-week + 12-week (*n* = 19) post DMM surgery. The coefficient of determination *R*^2^ and the *P*-values for each correlation coefficient are reported

Time point (weeks)	Thickness_PTA-CT_ vs Thickness_Histology_		Volume_PTA-CT_ vs Thickness_Histology_	
	*R*^2^	*P*-value	*R*^2^	*P*-value
4	0.8039	0.0011	0.9438	<0.0001
12	0.9354	<0.0001	0.6762	0.0035
4 + 12	0.8708	<0.0001	0.7606	<0.0001

However, comparing medial and lateral sides in either naïve or CTRL tibiae, we found that AC on the medial condyle was significantly thinner than on the lateral condyle [Fig. 4(E), (F)], a result that disproved our assumption of symmetry of AC along the tibial mid-coronal axis.

### 3D thickness maps of AC

In order to investigate on the axis of symmetry of AC, we generated thickness maps of the entire AC layer. The mediolateral and anteroposterior profiles of AC thickness (*n* = 4–6 samples per group) showed that the peak thickness was identical between medial and lateral side, confirming that symmetry between the condyles did exist. Maps of the CTRL and naive AC [Fig. 5(A), (B)] revealed that the location of the maximal AC thickness was not symmetric along the mid-coronal axis of the tibia but along a different axis tilted approximately by 12° from it. Moreover, the maximum thickness was located in the centre of each condyle and decreased radially away from the centre [Fig. 5]. Nevertheless, our automated ROIs [Fig. 5(A)] included the thicker zone of AC on both condyles and, despite generating asymmetric measurements, were considered adequate to evaluate AC loss. The AC thickness profiles of DMM samples evidenced a clear reduction in the thickness peak compared to CTRL (4-week post surgery) which was mostly contained within our automated ROIs.

### FE model of DMM-induced mechanical changes

The FE model was based on the 3D structure determined in the PTA-CT images of intact femoro-tibial joint joints [Fig. 6]. One axial loading event (no sliding) with the knee configured at a fixed flexion angle of 80° was simulated [Fig. 7(A)]. In order to focus on the effect of the structural changes in AC, and greatly simplify the model, the menisci and the altered joint mechanics induced by the DMM surgery were not included in the model. The contact regions, evidenced by the denser meshes, were centred over the areas of the maximal thickness of AC [Fig. 7(B), (C)], as displayed in the thickness maps [Fig. 5(A)]. The contact pressure on the tibial cartilage was elevated in the DMM compared with either CTRL or naïve (+39% and +45% respectively) [Fig. 7(D)]. An increase in contact pressure for a fixed displacement implies an increased local strain compared to normal levels.

Tresca stresses at the bone-cartilage interface increased in both tibia and femur in the DMM operated compared with either CTRL or naïve knees, as clearly visible in the sagittal views of the model (+43% and +53% respectively) [Fig. 7(E)]. Since Tresca stresses provide an estimate of the proximity to failure of a material under load, an elevation in local Tresca stresses at the bone-cartilage interface indicates an increased tendency of this tissue interface to permanently deform, i.e., to develop a structural lesion.

## Discussion

The current study demonstrates that the complete 3D geometry of the murine cartilage can be imaged by conventional microCT and segmented automatically. The key difference (and novelty), compared to recent similar studies^9^, ^18^, lies in the use of a conventional microCT scanner and in the unprecedented speed of the quantitative analysis of AC; in summary, a much increased throughput in the assessment of murine AC. We estimated that the total time required to assess AC by PTA-CT imaging is <10 h (8 h incubation in PTA, 1 h for scanning and reconstruction, 30 min for automated image analysis), compared to over 15 days using histopathology.

However, since PTA is toxic and requires long incubation times, PTA-CT cannot be extended to *in vivo* imaging. It should be also noted that, although we show that intact joints can be successfully imaged by PTA-CT [Fig. 6(C)], in the present study we split the joint and focused exclusively on the tibial cartilage. This is because other soft tissues surrounding AC (especially the menisci), also take up PTA [Fig. 6(C)] and cause errors in the automated segmentation of AC, thereby compromising analysis throughput. Nevertheless, conventional histological analysis has shown that most OA lesions in the murine DMM model are prevalent in the medial tibial epiphysis^28^, ^38^, ^39^, ^40^, supporting that notion that assessing OA in the tibia should provide a representative measure of OA in this model. Moreover, it is also possible to “virtually split” a joint by manual contouring a PTA-CT scan of an intact joint. Physical or virtual splitting of the joint will depend on users' needs or skills.

AC thickness values obtained by PTA-CT imaging were strongly correlated with histomorphometric thickness and in line with existing literature^2^, ^8^, ^9^, ^18^, ^41^. Our method, but not the histopathology score, showed statistically significant changes at 4-weeks post DMM surgery, suggesting that 3D quantitative measurements may achieve sensitivity compared to 2D histopathology scores, possibly owing to their reduced standard deviation. This conclusion is fully consistent with Ruan *et al.* (2013)^9^. Disease progression from 4 to 12 weeks after DMM surgery was confirmed by histopathology and our method accordingly.

To speed up and simplify the analysis, our software places automatically two ROIs in the load bearing area of each tibial condyle. This was motivated by previous histopathology studies^28^, ^38^, ^39^, ^40^ and, by our thickness maps of AC [Fig. 5], which, for the first time, have visualised the thickness distribution of AC on the mouse tibia.

Unlike ionic contrast agents^8^, PTA uptake in AC was not affected by changes in sGAG content caused be enzymatic digestion. This is a limitation of PTA-CT imaging, since sGAG loss is regarded as an early event at the onset of OA^8^, but also a strength, since AC segmentation is not affected by sGAG content.

Since calcified cartilage remained undistinguishable from SCB in PTA-CT images, it was not possible to determine whether the loss of AC thickness started from its surface or from an advancement of the tidemarks. However, the latter is unlikely in light of the recent finding that the calcified cartilage thickness does not significantly increase at 8 weeks post DMM^18^.

To our knowledge, this is the first study where FE modelling was employed to estimate the distribution of stresses in mouse AC whose 3D structure was directly obtained from segmented microCT scans. Whilst constituting a significant advancement in terms of resolution and realistic representation of the joints with respect to similar models in the literature^2^, our FE model should be considered an initial feasibility demonstration and, as such, presents several limitations. Firstly, it does not include the menisci and therefore does not take into account the altered joint mechanics due to DMM surgery, hence largely underestimating the increased loading on the medial side. Secondly, only one configuration is studied under compressive load and the full kinematics of the joint are not included in the simulations. Moreover, it does not consider: (1) the shear caused by the sliding surfaces on the joints during dynamic gait; (2) AC viscoelasticity and biphasic behaviour; (3) compositional changes induced by OA in AC and SCB (assigned constant, uniform properties). We can prove that this latter assumption is valid for bone since lowering bone elastic modulus from that of cortical bone (used in the present simulations) to that of homogeneous trabecular tissue had no significant effects on the simulations of the cartilage response (data not shown). However, the above limitations do not affect the outcome of this study due to its comparative nature. Importantly, as shown in Fig. 7, significant increases in contact pressure on the cartilage surface were co-localised with the regions of thickness loss in AC at early stages in the DMM model, which confirmed the focal and mechanical nature of OA pathogenesis. Consistently, contact pressures in AC were shown to increase as AC thickness decreases in another FE analysis where AC was modelled as a layer with arbitrary varying thickness^2^. However, any conclusion inferred from of our present FE simulations will need to be supported by more extensive investigations and data analysis.

In conclusion, we described a fast and simple method to visualise and automatically quantify 3D structural changes in adult mouse AC non-destructively. Once applied more extensively to confirm its robustness, this method may be used to increase screening throughput in the assessment of murine OA. The combined FE analysis might help linking genetic determinant of OA in murine models to cartilage mechanical changes, which will be crucial for the understanding of the pathogenesis of OA.

## Author contributions

• Conception and design: PDNB, TLV, DD, MM
• Collection and assembly of data: PDNB, AF, MM
• Analysis and interpretation of the data: all co-authors
• Drafting of article: PDNB, AF, MM
• Critical revision: PDNB, TLV, DD, MM
• Final approval of the article: all co-authors
• Statistical analysis: PDNB, MM
• Obtaining of funding: MM

## Role of the funding source

The founding source had no involvement in any part of the current study.

## Conflict of interest

All of the authors are employed by academic institutions and have no conflicts of interest to declare.
